# Less healthy breakfast cereals are promoted more frequently in large supermarket chains in Canada

**DOI:** 10.1186/s12889-017-4886-3

**Published:** 2017-11-13

**Authors:** Monique Potvin Kent, Erika Rudnicki, Crystal Usher

**Affiliations:** 1 School of Epidemiology and Public Health, Faculty of Medicine, University of Ottawa, 600 Peter Morand Cres. Room 301J, Ottawa, ON K1G5Z3 Canada; 2 Interdisciplinary School of Health Sciences, Faculty of Health Sciences, University of Ottawa, 25 University Private, Ottawa, ON K1N 6N5 Canada

## Abstract

**Background:**

The majority of food expenditures are made in supermarkets and this environment influences our purchasing and food intake. Breakfast cereals are frequently marketed as healthy food choices. The objective of this study was to examine the frequency of in–store promotions for cold breakfast cereals in Canadian supermarkets and to determine whether healthier or less healthy breakfast cereals are promoted more frequently.

**Methods:**

Data was collected once per week over a four-week period from a convenience sample of the five largest Canadian supermarkets in the Ottawa/Gatineau area. Data collection included the number of shelf facings, promotional displays, and the cost of cereals/100 g. The UK Nutrient Profiling Model was used to determine the healthfulness of each breakfast cereal.

**Results:**

29.8% (*n* = 67) of the 225 unique cereals were classified as healthier and 70.2% (*n* = 158) were classified as less healthy. Less healthy cereals were displayed at eye level, in the profitable middle shelves, 2.9 times more frequently than healthier cereals. There were 5.3 times more breakfast cereal shelf facings, 4.2 more end cap displays, 1.7 more mid-aisle displays and 3.3 more special pricing signage for less healthy cereals compared to healthier cereals. Less healthy cereals had a significantly higher average total number of shelf facings compared to healthier cereals (*t* = −4.28 (280.8), *p* < .001).

**Conclusions:**

Breakfast cereal manufacturers need to consider reformulation of their breakfast cereals to improve their healthfulness and supermarkets need to increase the marketing of healthy breakfast cereals within their stores.

## Background

Between 1980 and 2008, the worldwide prevalence of obesity almost doubled [1]. Today, more than one in nine Canadian children, as well as one in four Canadians adults are classified as obese [2, 3]. Globally, research shows increased consumption of unhealthy foods between 1990 and 2010 and, in developed countries such as the United States, Canada, Australia, New Zealand and Western Europe, the consumption of these foods is at the highest level worldwide [4]. The food intake of Canadian adults frequently does not meet nutritional guidelines. Half do not consume five servings of fruits and vegetables, and more than 25% exceed the Acceptable Macronutrient Distribution Range for fat [5].

Supermarkets, where the majority of food expenditures are made, influence our purchasing and consumption patterns [6]. In 2016, 73% of Canadians expenditures on food were made in grocery, specialty food, warehouse, and convenience-type stores [7]. The design of these stores, which contain more than 40,000 items, and the placement of these items influence our purchasing [6]. There is also evidence that in-store promotions in supermarkets influence food choices particularly since more than 2/3 of decisions regarding food purchases are made while grocery shopping [6, 8].

Breakfast cereals are frequently marketed as a healthy food option. Breakfast cereal packaging includes frequent health and nutrition claims, implying that the cereal is a healthy choice despite evidence to the contrary [9–12]. Other research has shown that breakfast cereals are heavily marketed to children on television and on the Internet and that the majority of these products can be classified as unhealthy [13]. While an analysis of child-targeted breakfast cereals sold in Canadian supermarkets indicated that 93% of breakfast cereals had high sugar content [14], no research has examined in-store promotions for cold breakfast cereals in supermarkets.

The objective of this research was thus to examine the frequency of in-store promotions for breakfast cereals in Canadian supermarkets; and, analyze whether less healthy breakfast cereals are being promoted more frequently than healthier breakfast cereals. It was hypothesized that less healthy breakfast cereals are promoted at a higher frequency than healthier breakfast cereals.

## Methods

### Data collection of promotions in supermarkets

The largest supermarkets in Canada (Loblaws, Sobeys (owned by Empire Co.), Metro, Costco, and Wal-Mart) were selected due to total sales [15]. Data collection took place over four consecutive weeks at a convenience sample of each of the selected supermarkets throughout Ottawa/Gatineau (in the provinces of Ontario and Quebec). Two undergraduate-level research assistants trained over a three-week period, collected data on four consecutive Thursdays or Fridays. All cold ready-to-eat breakfast cereals were recorded as often as they appeared in relation to their weight. For example, if Honey Nut Cheerios appeared in packages of 500 g, 750 g, and 900 g, all three were recorded as separate cereals. The total number of unique cereals was generated from the total list of all cereals by removing the duplicates of cereals with differing weights.

During the first week of data collection, general information about each cereal in the grocery store was recorded. This included the cereal name, company name, location of the cereal in the supermarket, shelf location, number of shelf facings, presence of end cap and mid-aisle displays, and price of the cereal (special pricing signage and price per 100 g). During the following three weeks, data collection consisted of collecting information on the number of shelf-facings, the presence of end cap and mid-aisle displays, and the price of the cereal (special pricing signage and price per 100 g).

The location of the cereal was recorded in week 1. Breakfast cereals were classified as being located in the main cereal aisle, health food aisle, club pack aisle (i.e. aisle for oversized items), another aisle of the supermarket, or located in two or more aisles. Shelf location of each cereal was also determined and it was recorded if the cereal was located on the top shelf, or bottom shelf. If the cereal was located on any of the shelves in between, the cereal was classified as being on the middle shelf and if the cereal was on multiple shelves it was recorded as such. The number of shelf facings was the number of times a cereal was positioned toward the aisle in any aisle in the supermarket, with the front of the cereal box directly aligned with the shelf as per the definition used by Chandon, Hutchinson, Bradlow, & Young [16]. Shelf facings were not counted if the cereal was pushed further back from the front of the shelf or if the front of the box was not positioned towards the aisle.

The number of end cap and mid aisle displays was determined in each week. End cap displays were cereal displays that were located at the end of the aisle in the grocery store. A mid-aisle display was a cereal display that was in the middle of the aisle or in an open area throughout the supermarket outside of the main aisle. These displays are typically utilized by supermarkets to promote in-store products and often feature sale items [17]. Cereals in end-cap and mid-aisle displays counted towards the total shelf facings for a cereal.

The original price and sale price (if applicable) of all breakfast cereals was recorded in week 1. The weight of the cereals was recorded in grams in order to calculate the price per 100 g. The sale price, if applicable, was recorded for all cereals for weeks 2, 3 and 4. It was assumed the cereals that were not on promotion for weeks 2, 3 or 4 had the same original price that was recorded for that cereal in week one. Finally, special pricing signage was recorded when the cereal had a visible price tag indicating that the cereal was on sale from the original price.

### Assessment of healthfulness of breakfast cereals

The healthfulness of breakfast cereals was determined using the UK Nutrient Profiling Model and each cereal was classified as healthier or less healthy. This model was developed by the Food Standards Agency and Ofcom to help determine what food and beverages could be marketed on television to children less than 16 years of age [18]. The model has been frequently used in research and has been shown to be a reliable and valid tool to assess the healthfulness of food and beverages [19, 20]. This scoring system allocates points based on the nutrient content of 100 g of the food product. The “A” points are awarded for total sugar, sodium, energy and saturated fat, while the “C” points are awarded for fruit, vegetable and nut content, protein as well as fibre. The “C” score is then subtracted from the “A” score, resulting in the total nutrient profile score. If the cereal’s total nutrient profile score is four or more points, it is classified as less healthy and if the score is three or below, the cereal is classified as healthier [21]. If cereal was sold in multi-packs (i.e. multiple small boxes of cereal in one larger pack), the nutritional information was not recorded. These cereals (*n* = 2) were excluded from the analyses.

### Statistical analysis

The data were inputted and analyzed with IBM SPSS Statistics Version 23 (2015). The frequency of breakfast cereals by healthfulness was calculated by breakfast cereal manufacturer and by supermarket. The total shelf facings per breakfast cereal over the four-week period for all five stores was calculated by summing the shelf facings for all four weeks for all of the five stores. An average was then calculated to obtain the average total shelf facings per cereal for all four weeks for all of the five stores. The average total end cap display, mid-aisle display, and special pricing signage for all four weeks for all five stores were calculated using the same methodology as the average total shelf facings.

To calculate the price per 100 g of the cereal in one week, the regular price of the cereal was divided by the total weight, in grams, of the cereal and multiplied by a factor of 100. In order to calculate the average cost per 100 g of each cereal over 4 weeks in 5 stores, the price per 100 g of each cereal was added together for all weeks at all five stores. The values were then divided by a factor of 20 (five stores multiplied by four weeks) to get an average price per 100 g for each week per breakfast cereal.

Comparisons between healthier and less healthy cereals were made using ratios. T-tests for independent samples were calculated when possible.

## Results

### Frequency of healthier and less healthy cereals by company and supermarket

In total, 305 cereals were recorded in 5 supermarkets though after eliminating cereals that were available in various package sizes, there were 225 unique cereals. Kellogg’s had the highest number of unique cereals (*n* = 50) followed by General Mills (*n* = 43) and Nature’s Path (*n* = 23). The total frequency of healthier and less healthy unique cereals by company across the five supermarkets is shown in Table 1. Overall, 29.8% (*n* = 67) of unique cereals were classified as healthier and 70.2% (*n* = 158) were classified as less healthy. Kellogg’s had the highest number of healthier cereals (*n* = 18; 26.9%), followed by President’s Choice (*n* = 11; 16.4%), and Empire Company (*n* = 7; 10.4%). General Mills had the highest number of less healthy cereals (*n* = 39, 24.7%), followed by Kellogg’s (*n* = 32, 20.3%) and Nature’s Path (*n* = 18, 11.4%). When the number of unique cereals by supermarket was examined, Metro had the highest number of cereals (*n* =144), followed by Sobey’s (*n* = 119), Loblaws (*n* = 117), Walmart (*n* = 83) and Costco (*n* = 18) (data not shown). At Costco, 88.2% of the unique cereals sold were classified as less healthy; whereas this percentage was 78.0% at Walmart, 74.8% at Metro, 73.9% at Sobey’s, and 72.4% at Loblaws.

**Table 1:** Frequency of unique cereals by company name in main cereal aisle and in health food aisle across 5 stores

Company name	Healthier *n* (%)	Less healthy *n* (%)	Total *n* (%)	Ratio of healthier to less healthy cereals
Kelloggs	18 (26.9)	32 (20.3)	50 (22.2)	1: 1.8
General Mills	4 (6.0)	39 (24.7)	43 (19.1)	1:9.8
Nature’s Path	5 (7.5)	18 (11.4)	23 (10.2)	1:3.6
President’s Choice	11 (16.4)	9 (5.7)	20 (8.9)	1: 0.8
Post	6 (9.0)	13 (8.2)	19 (8.4)	1:2.2
Metro	4 (6.0)	11 (7.0)	15 (6.7)	1:2.8
Empire Company Limited	7 (10.4)	6 (3.8)	13 (5.8)	1:0.9
Pepsi	2 (3.0)	10 (6.3)	12 (5.3)	1:5
ABF Grain Products Limited	1 (1.5)	11 (7.0)	12 (5.3)	1:11
Weetabix	5 (7.5)	0 (0)	5 (2.2)	n/a
Other companies^b^	4 (6.0)	9 (5.7)	13 (5.8)	1:2.3
Total	67 (100)	158 (100)	225 (100)	1:2.4

^b^Other category refers to Dorset cereal, Love Grown Foods, Price First, Wildroots and One Degree Organic Foods

### Frequency of healthier and less healthy cereals by shelf location

A total of 19.8% of cereals were displayed on the bottom shelf, 52.9% were displayed on the middle shelves, 24.5% were on the top shelf and 2.9% were found on multiple shelves (data not shown). Less healthy cereals were displayed at eye level, in the middle shelf, 2.9 times more frequently than healthier cereals.

### Shelf facings and other marketing techniques

Across all 5 stores and all 4 weeks there was a total of 3268 shelf facings as shown in Table 2. A total of 516 (15.8%) shelf facings consisted of healthier cereals and the remainder consisted of less healthy cereals (*n* = 2752, 84.2%). Therefore, there was 1 healthier breakfast cereal shelf facing for every 5.3 less healthy breakfast cereal facings. The cereals with the highest number of total shelf facings over the four week period for healthier and less healthy cereals are displayed in Table 3. The healthier cereal with the highest total number of shelf facings was Mini Wheats Original (1600 g) with 43 shelf facings. The less healthy cereal with the highest total number of shelf facings was Frosted Cheerios (340 g) with 96 total shelf facings.

**Table 2:** Total shelf facings and marketing techniques for all cereals for all four weeks for all five supermarkets

	Healthier cereals n (%)	Less healthy cereals n (%)	Total cereals n (%)	Ratio of healthier to less healthy cereals
Shelf facings	516 (15.8)	2752 (84.2)	3268 (100)	1: 5.3
End cap displays	13 (19.4)	54 (80.6)	67 (100)	1: 4.2
Mid-aisle displays	35 (36.5)	61 (63.5)	96 (100)	1: 1.7
Special pricing signage	138 (23.5)	450 (76.5)	588 (100)	1: 3.3

**Table 3:** Top 10 cereals with highest quantity of total shelf facings across four weeks across five stores

Ranking	Healthier cereals			Less healthy cereals		
	Cereal name	Company Name	Total Number of shelf facings	Cereal name	Company Name	Total Number of shelf facings
1	Mini Wheats Original (1600 g)	Kellogg’s	43	Frosted Cheerios (340 g)	General Mills	96
2	Shredded Wheat Original (425 g)	Post	25	Honey Nut Cheerios (685 g)	General Mills	78
3	Shreddies Granola Almond Crunch (540 g)	Post	23	Sunrise Crunchy Maple (750 g)	Nature’s Path	73
4	Life Multigrains (425 g)	Pepsi	22	Boo Berry (270 g)	General Mills	66
5	Special K Protein (800 g)	Kellogg’s	21	Shreddies Original (550 g)	Post	66
6	Oatmeal Crisp Triple Berry (650 g)	General Mills	20	Honey Nut Cheerios (460 g)	General Mills	65
7	Go Lean (370 g)	Kellogg’s	18	Raisin Bran (1150 g)	Kellogg’s	63
8	Mini Wheats Original (700 g)	Kellogg’s	18	Multigrain Cheerios (1180 g)	General Mills	60
9	Oatmeal Crisp Almond (710 g)	General Mills	18	Count Chocula (295 g)	General Mills	55
10	Go Lean Crunch (390 g)	Kellogg’s	16	Chocolatey Trix (330 g)	General Mills	52

With regard to the other marketing techniques examined, across all five stores and four weeks there were a total of 67 end cap displays, 96 mid-aisle displays and 588 instances of special pricing signage as shown in Table 2. For each marketing technique examined for healthier cereals, there were 4.2 end cap displays, 1.7 mid-aisle displays and 3.3 special pricing signage for less healthy cereals.

The average total shelf facings per cereal for four weeks for five supermarkets is presented in Table 4. On average, each cereal had a total of 6.0 shelf facings over four weeks and five supermarkets. Less healthy cereals had a significantly higher average total number of shelf facings over 4 weeks and 5 supermarkets (X = 12.23, SD = 15.74) compared to healthier cereals (X = 6.53, SD = 7.29; *t* = −4.28 (280.8), *p* < .001). No statistically significant differences were found among the average total number of other marketing techniques between healthier and less healthy cereals.

**Table 4:** Average total shelf facings and marketing techniques per cereal for all four weeks for all five supermarkets according to cereal healthfulness

	Healthier cereals	Less healthy cereals	Total cereals	t-test	
	X (SD)	X (SD)	X (SD)	t(df)	*p* value
Shelf facings	6.53 (7.29)	12.23 (15.74)	6.0 (14.24)	−4.28 (280.8)	0.001
End cap displays	0.16 (.52)	0.24 (0.69)	0.0 (0.65)	−0.88 (303)	0.382
Mid-aisle displays	0.44 (1.02)	0.27 (0.87)	0.0 (0.91)	1.35 (119.4)	0.181
Special pricing signage	1.75 (2.03)	1.99 (1.97)	1.0 (1.98)	−0.942 (303)	0.347

### Cost per 100 g

The average cost of all breakfast cereals per 100 g throughout the four weeks and in the five stores was $1.11 per 100 g as shown in Table 5. There were no statistically significant differences between healthier (X = 1.06, SD = 0.35) and less healthy cereals (X = 1.12, SD = 0.37; *t* = −1.28 (302), *p* = 0.2).

**Table 5:** Average cost ($) per 100 g of healthier and less healthy breakfast cereals over four weeks in five stores

Healthier cereals			Less healthy cereals			Total			t-test	
Mean (SD)	Min. cost per 100 g	Max. cost per 100 g	Mean (SD)	Min. cost per 100 g	Max. cost per 100 g	Mean (SD)	Min. cost per 100 g	Max. cost per 100 g	t(df)	*p* value
1.06 (0.35)	0.55	2.40	1.12 (0.37)	0.34	2.51	1.11 (0.37)	0.34	2.51	−1.28 (302)	0.2

## Discussion

In our study, not only were 70.2% of cold breakfast cereals sold in large supermarkets classified as less healthy, but the majority of marketing features within the supermarkets highlighted less healthy breakfast cereals as had been hypothesized. Less healthy cereals were displayed at eye level, in the profitable middle shelves, 2.9 times more frequently than healthier cereals. There were 5.3 times more breakfast cereal shelf facings for less healthy cereals compared to healthier cereals and less healthy cereals had a significantly higher average total number of shelf facings compared to healthier cereals (*t* = −4.28 (280.8), *p* < .001). Another significant finding was that there were 4.2 more end cap displays, 1.7 more mid-aisle displays, and 3.3 more special pricing signage for less healthy cereals compared to healthier cereals in our sample of supermarkets. Research has shown that all of these marketing features have an impact on sales. The shelf location of food products has been shown to influence customers’ decision to purchase certain items [22]. In particular, food products on the middle shelf are the most likely to be purchased because they are aligned with the customers’ line of sight [23, 24]. The quantity of shelf facings influences the likelihood that more attention is focused on those products [16] and research suggests that items with a higher quantity of shelf space can increase sales by 2% to 8% [25]. End cap displays have been found to substantially increase the likelihood of purchase of a given product and are typically filled with items on promotion [26, 27]. Prominent products that are placed in end cap displays account for approximately 30% of all supermarket sales [28, 29]. Mid-aisle displays are used for temporary products that are on promotion [30]. The placement of promotional food items in mid-aisle displays encourages the consumer to view the product. This suggests that consumers travelling throughout the supermarkets are much more likely to arrive at a less healthy cereal in a mid-aisle display than a healthier breakfast cereal. Finally, special pricing signage, utilized when a product is on sale or to promote a product within a store, prompts the consumer toward purchasing a product, whether or not the consumer originally wanted the item [31]. Given that there were a greater number of less healthy cereals at eye level, more shelf facings, end cap displays, mid-aisle displays, and special pricing signage for less healthy cereals, our result suggests that Canadian supermarkets are designed to encourage the purchase of less healthy cereals. It is unclear, given that there was a higher frequency of less healthy cereals, as to whether the differences in frequency in marketing techniques between the healthier and less healthy cereals were a result of randomly selecting cereals to promote, or a systematic bias toward promoting less healthy cereals in Canadian supermarkets. However, the fact that the ratio of healthier to less healthy cereals for shelf facings (1:5.3), end cap displays (1:4.2) and special pricing signage (1.3.3), outweighed the ratio of healthier to less healthy cereals (1:2.4), suggests that Canadian supermarkets may have a systematic bias toward promoting less healthy cereals. Given this imbalance in Canadian supermarkets where unhealthy breakfast cereals are promoted more frequently, public health officials and governments need to work with supermarkets to encourage the promotion of healthier food products. In addition, more research is needed to determine whether this imbalance in marketing exists for other product categories in the store.

One surprising finding in our research was that there was no significant difference between the prices of healthier or less healthy cereals. This result contrasts with the literature which suggests that less healthy food products are less expensive than healthier food options [32, 33]. A possible explanation for this finding is that grain products are the least expensive of all the food groups [32], therefore it can be hypothesized that the price gap between healthier and less healthy food items in this category is narrower than in other food categories.

Our results also indicate that breakfast food cereal manufacturers need to broaden their breakfast cereal category to include healthier cereals or, preferably, reformulate their products by reducing sugar and increasing fibre. The companies with the highest number of less healthy cereals included General Mills (*n* = 39), Kellogg’s (*n* = 32) and Nature’s Path (*n* = 18). General Mills also had 9.8 times more less healthy cereals than healthier cereals and Nature’s Path had one healthier cereal for every 3.6 less healthy cereals. It is evident that breakfast cereal manufacturers need to be encouraged to reformulate their products and broaden their product categories to include a higher number of healthier cereals. In the recently released program to combat childhood obesity in the United Kingdom, the government has encouraged food and beverage companies to decrease sugar content in nine product categories by 20% by 2020 [34]. If such an approach was adopted in Canada, the healthfulness of breakfast cereals would likely be improved.

Large supermarkets also need to consider the healthfulness of the products stocked on their shelves. At Costco, only one healthier breakfast cereal was available on their shelves compared to 13 less healthy cereals while at Walmart there were 4.3 less healthy cereals for every healthier cereal. Moving healthier cereals to the middle shelf at eye level will make customers notice these cereals and increase the likelihood that they will be purchased. Increasing the number of healthier cereals in promotional locations such as end-cap and mid-aisle displays is also recommended to make the cereals more visible. Bright, colorful, large special pricing signage for healthy breakfast cereal sales could also be used in order to attract customers to these cereals. Multi-pronged interventions that have increased the supply and in-store promotion of healthier foods have led to increased purchases of healthier foods in small retail environments [35] though interventions in large supermarkets have had mixed results [36]. Since the 1970’s, large supermarkets have been selling shelf space to retailers called slotting fees. According to research conducted by the U.S. Federal Trade Commission [37], retailers negotiate with suppliers on a variety of fees which include slotting fees. As a result, these fees can vary between and within product categories. While slotting fees permit access to shelf space in grocery stores, the exact placement of the product is not typically guaranteed and the specific location of the product is often determined by product demand and profit level [37]. Based on this research, one can assume that either less healthy cereals have a higher profit margin and/or that they are in greater demand than healthier cereals. While research needs to be conducted in other product categories, methods to incentivize large supermarkets to promote healthier products need to be developed particularly since research has shown that availability of healthier products is a means to encourage the purchase of healthier products [38]. Clearly, the status quo is leading to an obesogenic environment within the cereal aisle.

### Strengths and limitations

This study is the first to assess the frequency of  healthier and less healthy breakfast cereal promotions in  Canadian supermarkets. Data collection took place in the five largest supermarket chains by sales in Ontario and Quebec which have the highest population density in Canada. Data was collected over four weeks on the same day of the week for consistency and a rigorous validated system was used to classify breakfast cereals as healthier or less healthy. Limitations to the study include that the price for Week 1 was substituted for the price at Weeks 2–4 for cereals that were not on promotion during these weeks. We were unable to control for grocery store size as square footage was not available for all participating supermarkets. Also, a convenience sample of grocery stores was used rather than a random sample which impacts the generalizability of this study. Replication with a random sample of grocery stores in Canada is warranted. Our research only focused on one product category (breakfast cereals) and it cannot be assumed that the results apply to other product categories. More research is needed that focuses on the marketing of healthier and less healthy foods and beverages within the entire supermarket. In addition, as this study focused exclusively on measuring promotions within the supermarket environment, research that considers the impact of supermarket environments that promote less healthy cereals more frequently than healthier cereals on consumers is recommended.

## Conclusions

Less healthy breakfast cereals are promoted more frequently in large supermarket chains in Canada and changes are clearly needed in this environment in order to encourage the purchase and consumption of healthier breakfast cereals. While large supermarkets need to consider the healthfulness of the products stocked on their shelves, other stakeholders also need to work on changing this environment. Breakfast food cereal manufacturers need to reformulate current products and broaden their breakfast cereal offerings to include healthier cereals. Public health officials and governments also need to work with supermarkets to encourage the promotion of healthier food products and consider the development of methods to incentivize large supermarkets to promote healthier breakfast cereals.
